# The Moderating Effect of Cultural Intelligence on the Relationship Between Emotional Intelligence and Job Satisfaction

**DOI:** 10.3389/fpsyg.2022.900546

**Published:** 2022-05-30

**Authors:** Yasemin Bal, Özgür Kökalan

**Affiliations:** ^1^Department of Business Administration, Yıldız Technical University, Istanbul, Turkey; ^2^Department of Business Administration, Istanbul Sabahattin Zaim University, Istanbul, Turkey

**Keywords:** cultural intelligence, emotional intelligence, job satisfaction, academicians, Turkey

## Abstract

It is seen that employees with high emotional intelligence (EI) generally have low level of job stress; they can also integrate better with their jobs and provide a high level of job satisfaction (JS). This study aims to investigate the moderating effect of the cultural intelligence (CI) level of academicians on the relationship between their EI and JS. The data were collected from 470 academicians working in Turkish universities. The sample consists of 3 sub-groups: academicians that are Turkish citizens of and completed their entire education in Turkey, academicians that are Turkish citizens and received a part of their education abroad, and academicians that are not citizens of Turkey and completed their education outside of Turkey. According to the research results, it was found that there is a significant positive relationship between the EI level of the academicians and their JS. The research also determined that CI had a moderating effect on the positive relationship between EI and JS. The CI level of the academicians strengthens the positive relationship between their EI and JS.

## Introduction

The increasing globalization of business life has brought some managerial requirements and difficulties with it. The increase in the number of global managers and expatriates working across borders and the increase in international circulation have caused issues such as EI and CI to gain importance. Being able to adapt to new cultural environments, establishing healthy and effective communication with people from different cultures, and managing employees with intercultural empathy have become the most basic characteristics that today’s managers should have. EI and CI have become some of the most sought-after features in recent years, not only for managers but also for employees and students. It is observed that people with high EI can be happier, more peaceful, and calm in both their social lives and work lives. These people can overcome stress more easily in their business life, can empathize, and their JS levels become higher. It is seen that employees who can manage their emotions and adapt to cultural changes are more satisfied with their work lives.

Many researches are carried out on CI and EI, and the reflections of these variables on business life. One of the most researched concepts in this field is the effect of these variables on the JS levels of the employees. The relations of these variables with JS and the research examples in the literature are discussed in detail in the relevant sections below. In the literature, there are studies investigating the effects of individuals’ EI and CI levels on JS separately. In this study, the effects of the coexistence of these two variables on JS were examined. For this reason, the moderator analysis method was used. It is expected that our study will fill an important gap in the field and will guide further studies. This study will also provide insights to academicians working in the relevant field and practitioners in business life.

## Literature Review

### Emotional Intelligence

EI originates from the social intelligence concept that was introduced by Thorndike (1920). Thorndike used the term “social intelligence” to explain the skill to perceive and manage other people. Later, Gardner (1983) defined multiple intelligences and stated that intrapersonal and interpersonal intelligence are as important as IQ. Salovey and Mayer (1990) defined EI as the ability to understand and monitor one’s own and others’ emotions, to distinguish between emotions, and to use this information to guide thought and action. Then the term EI became popular with the publication by Goleman (1995). Goleman (1995) defined EI as “a skill which a person can manage others’ moods through self-awareness, improve it through self-management, comprehend its effect through empathy and behave in a way that increases others’ morale through effective relationships.” EI is expressed as the sum of cognitive abilities that allow people to perceive, identify, evaluate, interpret, and manage emotional information about themselves and others (Salovey and Mayer, 1990; Mayer et al., 2016).

Mayer and Salovey (1997) later expanded their EI definition and included the capacity to perceive and absorb feelings, understand information about these emotions, and manage them. The authors defined four dimensions of EI. These dimensions were identified as emotional self-awareness, emotional other-awareness, emotional self-regulation, and using emotion to facilitate performance. Based on Salovey and Mayer’s model of EI, Schutte et al. (1998) developed a 33-item EI scale. The scale developed by Schutte et al. (1998) consists of 4 dimensions; empathic sensitivity, positive regulation, positive utilization, and emotional appraisal. Empathic sensitivity reflects an individual’s general sensitivity or empathy to the emotional expressions of others. Positive utilization describes the individual’s positive use of emotions in a way that allows for the evaluation and generation of new ideas. Positive regulation refers to the individual’s positive outlook and expectations in mood regulation. Emotional appraisal reflects awareness, perception, understanding, and experience of an individual’s own feelings.

EI has been accepted as a powerful factor in both employee performance and organizational performance. It also makes a positive effect on work team performance. Teams whose members and leaders have a strong EI can be more creative, successful, and innovative in the workplace. People with high EI know themselves very well. At the same time, they can perceive other people’s emotions and empathize. They are expressive, flexible, and optimistic. They can easily adapt themselves to the new and changing conditions. When individuals develop their EI, they can become more effective and successful in what they do and at the same time support other people to be more effective and successful. EI development also reduces the stress of both individuals and businesses by softening the conflicts seen in the workplace. Thus, it emphasizes understanding in human relations and encourages harmony (Serrat, 2009). In the last decades, EI seems like an important predictor of leader effectiveness. A leader’s high EI also has an influence on the success of his/her subordinates (Miao et al., 2016). Goleman (1998) emphasizes the role of EI in the success of leaders and states that successful leaders have high EI. EI can enhance leaders’ competency to overcome and solve problems that they face in business life (Liu and Liu, 2013). At the same time, managers use EI in their interpersonal communications to create an environment where employees are satisfied and motivated by their work (Udod et al., 2020). Considering the challenges of business life and managing people, it is obvious how important it is for a leader to have this feature.

### Cultural Intelligence

CI is closely related to EI. CI is one of the main factors that enable people to adapt to social life, work in harmony, and communicate with people from different cultures. Peterson (2004) stated that it is difficult to integrate into another culture and people trying to adapt to a new culture need both EI and CI. Today, the concept of CI, which has started to attract more attention, especially with globalization, has become a very important issue both for managers working across borders and for the education of new generations. With the increase in globalization, the rate of working in different countries and cultures has increased. In addition, people employed in multinational enterprises have to work in harmony with people from different cultures. Cultural diversity can serve as a challenge for especially expatriates and global managers because not all of them have the competence to interact effectively with different cultures (Bhaskar-Shrinivas et al., 2005; Ang and Van Dyne, 2008; Crowne, 2008). Conflicts, misunderstandings, communication problems, and cultural tensions may occur due to cultural differences, especially in multinational enterprises. Some individuals may perform better than others in a cross-cultural work environment (Brancu et al., 2016). They can adapt to the changing cultural conditions easily and express themselves in different cultures. The main reason for this is that these people have higher CI. Investigating CI can be important to determine how global managers cope with challenges and succeed in the global business environment (Barakat et al., 2015).

The CI concept is developed and searched in various studies in the literature. CI is defined as “a person’s ability to adapt successfully to unfamiliar environments and new cultural environments related with the cultural context” (Earley and Ang, 2003, p. 26). Peterson (2004) defined CI as the ability to use one’s skills appropriately in a cross-cultural setting. Goleman (1998) stated that a critical element shared by CI and EI is the tendency to suspend judgment and to think before you act. *S*uspension may take hours or days for a person with a high level of CI, while it may take weeks or months for someone with a low level of CI (Earley and Mosakowski, 2004a,b).

Ang et al. (2007) defined four dimensions of CI as meta-cognitive, motivational, cognitive, and behavioral. Meta-cognitive, cognitive, and motivational CI dimensions reflect the mental capabilities of an individual. Meta-cognitive CI refers to the mental processes a person must have to understand and analyze information (Ang et al., 2007). It is defined as a person’s conscious cultural awareness and capacity to process information, including intercultural encounters (Ang and Van Dyne, 2008). Cognitive CI includes a person’s level of knowledge about the norms, values, and practices of any given culture. These cognitional competencies are especially essential for international experience and education. Motivational CI reflects the desire to initiate, direct, and sustain energy to learn and adapt to unusual situations and tasks (Ng et al., 2012; Barakat et al., 2015). Behavioral CI describes an individual’s verbal and non-verbal ability to communicate efficiently with people from different cultures (Earley and Ang, 2003). The CI scale constructed by Ang et al. (2007) is the most frequently used scale in studies on cultural intelligence. In our study, this scale was also used in the measurement of CI.

There are many studies with university students, global managers, and expatriates in CI literature. EI and CI are predictors of international adaptation success as Wakeman (2009) stated. Brancu et al. (2016) investigated the CI dimensions and tried to find out which dimensions could be observed more or less among university students. The study of Thompson (2018) revealed that EI and CI are highly effective in enabling international students to integrate and adapt socially. The results of some empirical studies in the field of business are as follows. Gorji and Ghareseflo (2011) found a positive relationship between EI, CI, and employee performance. Fu (2014) found that EI had a significant positive impact on the ethical behavior of respondents. Rafiq et al. (2020) focused on the moderating role of CI between emotional labor and emotional exhaustion. The results reveal that CI is a positive resource that can be used by employees to successfully regulate their emotions in culturally diverse organizational environments (Tay et al., 2008; Rafiq et al., 2020). Liao et al. (2021) investigated the influence of EI and cultural adaptability on cross-cultural adjustment with a sample of expatriates. The results of the study revealed the positive effect of EI and cultural adaptability on cross-cultural competence.

### The Relationship Between Job Satisfaction, Emotional Intelligence, and Cultural Intelligence

JS is defined as the general attitude and positive emotional state that reflects the individual’s emotional reaction, evaluation of the job experience, and carries the meaning of job success (Locke, 1976). JS can be described as individuals’ self-satisfaction about their work (Luthans, 1992). It is also defined as “an evaluative state that expresses satisfaction and positive feelings about one’s job” (Judge and Kammeyer-Mueller, 2012, p. 346).

JS is a concept that has been searched in the business literature with many different variables. Especially in the last decades, one of the variables frequently examined both theoretically and empirically in the studies on EI and CI is related to JS. JS of global managers is closely related to their cultural adaptation or fit, which is a result of CI. Self-efficacy, which is an antecedent of CI and JS, is seen as a very important factor for international managers to cope with and adapt to cultural problems they may face in the international era (Bandura, 2002). Higher EI is related to general psychological wellbeing, better job performance, organizational commitment, and JS (Wong and Law, 2002; Brackett and Mayer, 2003).

Some studies dealing with the relationships between JS, EI, and CI variables are as follows. Empirical researches show that employees with high EI generally have a low level of work stress. They can also integrate better into their job and perform high levels of JS (Slaski and Cartwright, 2002). Wong and Law (2002) found that supervisors’ EI was positively related to the JS of their subordinates. Sy et al. (2006) examined the relationship between JS and EI and they revealed that there was a positive relationship between EI, JS, and the performance of both managers and employees. Park et al. (2011) found that employees with high EI had high JS and it directly affected their productivity. Liu and Liu (2013) found that the leader’s EI had a stronger effect on members’ JS. Barakat et al. (2015) investigated the moderator role of JS on the relationship between CI and performance. It was stated that JS transferred the effect of CI to job performance. It was also revealed that global managers with high CI feel more JS and perform better in their jobs in the international business environment. Miao et al. (2016) found that subordinates’ EI was positively related to leaders’ EI and mediated the relationship between leaders’ EI and subordinates’ JS. EI had a stronger impact on subordinates’ JS because as a result of EI, subordinates could interpret leaders’ effective leadership behaviors as more willing and altruistic. Diemer (2016) examined the relationship between CI and work outcomes of Chinese expats. As a result of empirical analysis, a positive relationship was found between JS and CI of expatriates. Huang (2016) revealed that employees with high levels of EI showed a stronger positive relationship with JS. In their study, Telli and Zehir (2016) revealed a positive relationship between CI and JS and also they observed that high CI increased JS levels of global managers. Alam and Zaheer (2021) investigated EI as the moderator of the relationship between empowering leadership and burnout. They found that the association of empowering leadership and employee burnout was moderated by EI. As indicated by Szczygiel and Mikolajczak (2018), EI provides buffering from burnout. The study of D’Amico et al. (2020) showed that perceived EI positively correlates with work engagement and JS, and negatively correlates with burnout. In troubled situations, a worker with a high level of EI can handle problems and overcome stress easier than others by adapting easily to changing conditions (Alam and Zaheer, 2021). Lu et al. (2021) searched the effect of EI on the relationship between emotional labor and JS. Individuals with high levels of EI could code and decode their personal and other people’s emotions and manage their emotional behaviors by adapting to their work’s emotional demands. Alferaih (2021) revealed that EI positively impacted the performance and JS level of employees where engagement mediated the relationship between EI and JS. The findings of Bano et al. (2021) revealed that all dimensions of EI showed significant positive correlation with JS. Akhter et al. (2021) showed that EI and CI had a positive effect on bank employees’ JS levels. Schlaegel et al. (2022) found that EI dimensions were related to JS and investigated the structure of the EI-JS and EI-job performance relationship.

Although there are many studies with different variables in the related field, there is no study in the literature that examines the moderating effect of CI on the relationship between EI and JS. The study is important in terms of showing the difference between the EI and CI levels of academicians studying and working in Turkey and academicians studying or living abroad and working in Turkey. For this reason, this study fills an important gap in the empirical field in the relevant literature and contributes to the field. The following hypotheses have been generated based on the literature.

*Hypothesis 1(H_1)_: Emotional intelligence (EI) is positively associated with job satisfaction (JS).*
*Hypothesis 2(H_2_): Cultural intelligence (CI) moderates the effect of emotional intelligence (EI) on job satisfaction (JS).*

## Materials and Methods

### Sampling

Data were collected from academicians working in Turkish universities. In 2021, the total number of academicians in Turkey was reported as 98.404 (Higher Education of Turkey [YOK], 2021). The data were gathered from three types of academicians. The first type of group consists of academicians that are citizens of the Republic of Turkey and completed their entire education in Turkey. The second type of group consists of academicians that are Turkish citizens and received a part of their education abroad. The third type of group consists of foreign academicians that are not citizens of Turkey and completed their education outside of Turkey.

In the data collection process, participants were contacted via e-mail and their consent was obtained. All data were collected during 5 months period between January and June 2021. The online questionnaires were directly sent to participants’ e-mail account. The research was carried out on academicians working at universities in Turkey’s three big cities. At this stage, a questionnaire was sent to all foreign academicians working at universities in these three cities, since there were a limited number of them. Among the Turkish faculty members, approximately 2,000 faculty members selected randomly and were sent an e-mail. In the selection of the participants, the information of the academicians on the web pages of the universities and the Turkish higher education database were used.

The questionnaire was distributed three times to decrease common method bias (Podsakoff et al., 2012). In this process, a minimum 1-month time lag was taken to protect the biasing effects of occasional factors (Matthews et al., 2014). The questionnaire included four different parts. Demographic characteristics were asked to academicians in the first part. In the other parts, the CI, EI, and JS scale questions were asked, respectively, to the participants. At the end of 5 months period, 470 questionnaires were gathered from academicians.

The sample consisted of 254 women (54.04%) and 216 men (45.96%) with ages ranging from 25 to 65. It is seen that 356 (75.74%) of the participants are Turkish, 114 (24.26%) of them are foreigners. 50% of the participants know at least 2 foreign languages. It is determined that 120 (25.53%) of the participants were research assistants, 236 (50.21%) assistant professors, 38 (8.09%) associate professors, and 76 (16.17%) professors. It is also seen that 169 (35.95%) of the participants are Turkish academicians and have no experience of living abroad for more than 6 months; 187 (39.79%) are Turkish academicians and have experience of living abroad for more than 6 months, and 114 (24.26%) are foreign academicians working at different universities in Turkey.

### Measure

The questionnaire consisted of scales. The “Cultural Intelligence Scale (CIS)” developed by Ang et al. (2007) and validated in Turkish by Ilhan and Çetin (2014) was used. The CIS constructed by Ang et al. (2007) is the most frequently used scale in studies on CI. The scale aims to measure four different dimensions of CI called “Cognitive,” “Meta Cognitive,” “Behavioral,” and “Motivational.” CIS has 20 items. Sample items include “I know the cultural values and religious beliefs of other cultures,” and “I enjoy living in cultures that are unfamiliar to me.” The Cronbach Alpha score of CIS was found as 0.81.

The 33-item “Emotional Intelligence Scale (EIS)” was developed by Schutte et al. (1998). EIS consists of 4 dimensions; empathic sensitivity, positive regulation, positive utilization, and emotional appraisal. The short version of EIS with 12 items was used in this study. Sample items include “I feel fairly satisfied with my present job,” and “I find real enjoyment in my work.” The Cronbach Alpha score of EIS was found as 0.79.

The “Job Satisfaction Scale (JSS)” was developed by Agho et al. (1992). It consists of 6 items. Sample items include “I enjoy my job” and “I rarely get bored with my job.” The Cronbach Alpha score of JSS was found as 0.84. Responses were rated on a five-point Likert scale from “strongly disagree” to “strongly agree.”

### Statistical Analysis

The hypotheses were examined with Covariance based structural equation modeling (CB-SEM). In this process, AMOS version 25 was used. The normality of the data was firstly controlled. According to kurtosis and skewness scores of the data, it was seen that the data used in the study were normally distributed (Meydan and Şeşen, 2015). In the second step, factor structure and validation of the scales were assessed by confirmatory factor analysis (CFA). In this process, root-mean-squared error of approximation (RMSEA), chi-square test (χ^2^), adjusted goodness of fit index (AGFI), goodness of fit index (GFI), and comparative fit index (CFI) were taken as fit indices. The fitted value, greater than 0.90 for CFI and GFI, greater than 0.85 for AGFI and less than 0.10 for RMSEA, were taken (Sümer, 2000; Meydan and Şeşen, 2015). In addition to this, Harman’s single-factor test, the independent sample *t*-test, the one-way ANOVA test, and correlation analysis were analyzed by SPSS 25 program.

## Results

### Data Analyses

In the first step, common method variance was evaluated by Harman’s single-factor test. The first factor explained 24.65% of the total variance. It was seen that there was no common method bias. The descriptive information about variables was given above in Table 1.

**TABLE 1:** Descriptive statistics.

	Mean	SD	AVE	MSV	CR	Skewness	Kurtosis	1	2	3
1.Cultural intelligence (CI)	3.81	0.46	0.54	0.48	0.81	−0.33	0.96	1		
2.Emotional intelligence (EI)	4.01	0.49	0.58	0.48	0.79	−0.55	1.43	0.50**	1	
3.Job satisfaction (JS)	3.96	0.69	0.66	0.42	0.84	−0.76	0.88	0.31**	0.49**	1
5.Gender	1.46	0.49						0.00	−0.05	0.04
6.Age	37.3	7.36						0.10*	0.02	0.18*
7.Number of foreign language	1.71	0.77						0.18**	0.11*	0.04
8. Overseas life experience	1.88	0.76						0.05	0.22**	0.16**

*N = 470. SD, Standard Deviation; AVE, Average Variance Extracted; MSV, Maximum Shared Squared Variance; CR, Composite Reliability.*

**Correlation is significant at the 0.01 level (2-tailed).

*Correlation is significant at the 0.05 level (2-tailed).

The average score on the CI (*M* = 3.81; *SD* = 0.46) and EI (*M* = 4.01; *SD* = 0.49) were calculated. A significant difference was found between the CI levels of academicians living only in Turkey and academicians living both in Turkey and abroad (*t* = −2.51, *p* = 0.00). The CI level of academicians living both in Turkey and abroad was found to be higher than that of academicians living only in Turkey. The one-way ANOVA test results determined that no statistically significant differences was found (*F* = 1.57, *p* > 0.05; *F* = 1.86, *p* > 0.05). Significant differences were found in CI according to the number of languages an academic could speak. It has been determined that academicians who know more foreign languages have higher CI (*F* = 9.46, *p* < 0.01).

The JS mean and standard deviation value was found as 3.96 and 0.69. According to the independent sample *t*-test result, no statistically significant difference was found between male and female (*t* = −0.96, *p* > 0.05). According to ANOVA results, it was seen that significant differences in JS scores was found in terms of the academic status of the participants (*F* = 7.38, *p* < 0.01). It was determined that as the academic status increased, job satisfaction increased.

According to correlation analysis, it was determined that there is a significant positive relationship between CI and EI (*r* = 0.50, *p* < 0.01), and CI and JS (*r* = 0.31, *p* < 0.01). It was also determined that EI and JS are positively correlated (*r* = 0.49, *p* < 0.01).

According to the results of CFA, the model had an acceptable goodness of fit (RMSEA = 0.07; χ^2^ = 97.67; df = 25; *p* < 0.01, GFI = 0.95, CFI = 0.94, AGFI = 0.91).

#### Hypotheses Testing

H_1_ and H_2_ constructed within the scope of the study were analyzed with the AMOS program. According to the analysis results;

H_1_ proposes that EI is positively associated with JS. In Table 2, it was reported that EI is positively associated with JS (β = 0.63, SE = 0.05, *t* = 11.56, *p* < 0.01). H_1_ is supported.

**TABLE 2:** CI moderating analysis.

	Job satisfaction			
	H_1_		H_2_	
	β	SE	β	SE
Constant	0.92**	0.24	0.92**	0.24
Gender	0.03	0.02	−0.04	0.03
Age	0.04	0.04	0.00	0.09
Number of foreign language	0.00	0.01	0.03	0.10
Overseas life experience	0.03	0.02	0.05	0.03
Academic status	0.10**	0.02	−0.34	0.17
Emotional intelligence (EI)	0.63**	0.05	0.59**	0.09
Cultural intelligence (CI)			0.24*	0.07
EI *CI			0.10**	0.02
*R* ^2^	0.29		0.37	
F	95.94**		110.52**	

*N = 470. β, Beta Coefficient; SE, Standard Error; H_1_, Hypothesis 1; H_2_, Hypothesis 2.*

**Significant at the 0.01 level (2-tailed).

*Significant at the 0.05 level (2-tailed).

**FIGURE 1:**
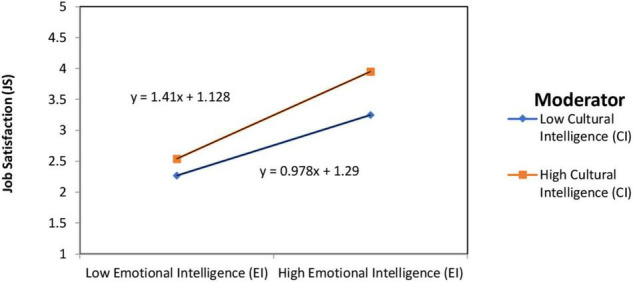
The interaction between EI and CI.

H_2_ proposes that CI moderates the effect of EI on JS. In Table 2 it was reported that the interaction term was statistically significant (β = 0.10, SE = 0.02, *t* = 4.90, *p* < 0.01). According to the result, it was determined that CI strengthens the positive relationship between EI and JS. This relationship was shown in Figure 1. H_2_ is also supported.

## Discussion and Conclusion

Although studies are investigating the relationship between EI, CI, and JS in the literature, there is no study to determine CI as a moderator of the relationship between EI and JS. Because of this reason, we examine whether CI is a moderator in the relationship between EI and JS. In this research, H_1_ proposes that EI is positively associated with JS. According to the analysis results it is found that EI is positively associated with JS and H_1_ is supported. H_2_ proposes that CI moderates the effect of EI on JS. It is found that the interaction term is statistically significant and H_2_ is also supported. CI moderates the effect of EI on JS, such that an academician with high levels of CI would be more likely to have more JS than an academician with less CI.

It is known that EI and CI, whose importance has increased with the effect of globalization in recent years, positively affect many outputs such as JS, motivation, creativity, and job performance. It is seen that in the literature, many studies support that EI is positively correlated with JS. When the employees develop their EI, they can become more effective. Higher EI is related to general psychological wellbeing, JS, better job performance, and organizational commitment (Wong and Law, 2002; Brackett and Mayer, 2003). EI also moderates the relationship between empowering leadership and burnout (Slaski and Cartwright, 2002; Park et al., 2011; Huang, 2016; Szczygiel and Mikolajczak, 2018). Individuals with high EI can better understand their own and others’ emotions and behave more optimistically. In that way, they can cope with the difficulties and stress in the work environment more easily and have more JS. Therefore, EI is of great importance in increasing both individual and organizational performance. CI also has come to the forefront as a very important concept in recent years, especially for people working in international businesses, people living or studying abroad, and managers of international businesses. There is a positive relationship between EI, CI, and employee performance. A high level of CI has a positive effect on employee performance and JS. EI should be one of the issues that managers should pay attention to, especially in increasing employee performance and JS.

Our research results reveal the importance of EI and CI, and it has been observed that CI in particular strengthens the relationship between JS and EI. The study is important in terms of showing the difference between the EI and CI levels of academicians studying and working in Turkey and academicians studying or living abroad and working in Turkey. Academicians were included as a sample in this study, but in future studies, the moderating role of CI could be investigated for different sectors, occupations, and variables by increasing the number of samples. This will allow us to see the relationships between the variables and especially the moderator effect of CI in more detail. Thus, the studies will contribute to both practitioners and managers in the field of business and scientists working in the related field.

## Data Availability Statement

The raw data supporting the conclusions of this article will be made available by the authors, without undue reservation.

## Ethics Statement

Ethical review and approval was not required for the study on human participants in accordance with the local legislation and institutional requirements. The participants provided their written informed consent to participate in this study.

## Author Contributions

YB wrote the literature part of the study. ÖK carried out the data collection process and made the analysis. Both authors discussed the findings of the article and finalized the article.

## Conflict of Interest

The authors declare that the research was conducted in the absence of any commercial or financial relationships that could be construed as a potential conflict of interest.

## Publisher’s Note

All claims expressed in this article are solely those of the authors and do not necessarily represent those of their affiliated organizations, or those of the publisher, the editors and the reviewers. Any product that may be evaluated in this article, or claim that may be made by its manufacturer, is not guaranteed or endorsed by the publisher.
